# Detection of Multiple Variants of Grapevine Fanleaf Virus in Single *Xiphinema index* Nematodes

**DOI:** 10.3390/v11121139

**Published:** 2019-12-10

**Authors:** Shahinez Garcia, Jean-Michel Hily, Véronique Komar, Claude Gertz, Gérard Demangeat, Olivier Lemaire, Emmanuelle Vigne

**Affiliations:** 1 Unité Mixte de Recherche (UMR) Santé de la Vigne et Qualité du Vin, Institut National de la Recherche Agronomique (INRA)-Université de Strasbourg, BP 20507, 68021 Colmar Cedex, France; shahinez.garcia@inra.fr (S.G.); veronique.komar@inra.fr (V.K.); claude.gertz@inra.fr (C.G.); gerard.demangeat@inra.fr (G.D.); olivier.lemaire@inra.fr (O.L.); 2 Institut Français de la Vigne et du Vin (IFV), 30240 Le Grau-Du-Roi, France

**Keywords:** GFLV, variants, *Vitis vinifera*, *Xiphinema index*, acquisition, High Throughput Sequencing (HTS), detection, Restriction Fragment Length Polymorphism (RFLP), genetic diversity, bottleneck

## Abstract

Grapevine fanleaf virus (GFLV) is responsible for a widespread disease in vineyards worldwide. Its genome is composed of two single-stranded positive-sense RNAs, which both show a high genetic diversity. The virus is transmitted from grapevine to grapevine by the ectoparasitic nematode *Xiphinema index*. Grapevines in diseased vineyards are often infected by multiple genetic variants of GFLV but no information is available on the molecular composition of virus variants retained in *X. index* following nematodes feeding on roots. In this work, aviruliferous *X. index* were fed on three naturally GFLV-infected grapevines for which the virome was characterized by RNAseq. Six RNA-1 and four RNA-2 molecules were assembled segregating into four and three distinct phylogenetic clades of RNA-1 and RNA-2, respectively. After 19 months of rearing, single and pools of 30 *X. index* tested positive for GFLV. Additionally, either pooled or single *X. index* carried multiple variants of the two GFLV genomic RNAs. However, the full viral genetic diversity found in the leaves of infected grapevines was not detected in viruliferous nematodes, indicating a genetic bottleneck. Our results provide new insights into the complexity of GFLV populations and the putative role of *X. index* as reservoirs of virus diversity.

## 1. Introduction

One of the main characteristics of RNA viruses is their high mutation rates. This is due to their highly error-prone RNA polymerase that produces mistakes during replication [1], stimulating the generation of divergent genotypes per infected cell and per cycle that surround a consensus genome sequence [2]. The dynamics of such highly polymorphic population as well as recombination, and reassortment enable the virus to adapt to ever-changing environments in the host and the vector, leading to selection/emergence of specific viral variants. Those elements as well as mutation, migration, selection, and genetic drift are the main driving forces in virus evolution [3].

Bottleneck contributes also to the evolution of plant virus populations. A bottleneck consists of a sharp reduction at the population (quantitative) or at the genetic (qualitative) levels. Bottlenecks can arise within a host-cell during various steps of the infectious cycle and during colonization of host organs by the virus or by grafting in the case of grapevine [4]. Bottlenecks can also occur from abiotic events such as deep freeze or drought and when a perennial plant is harvested and pruned at the end of the growing season. In addition, shift in viral populations can happen during host-to-host horizontal transmission by a vector [5]. Genetic variation caused by vector transmission from an infected source plant host to a newly infected plant host, can appear during three distinct steps: (i) the acquisition of the virus by the vector by ingestion, (ii) the retention/adsorption of some particles by the vector, underlying an active process where transmissible particles are retained (for a certain amount of time) at specific sites within the feeding apparatus, while others pass through the oesophagus into the intestine and are digested, and (iii) the inoculation phase which allows the release of virus particles from specific vector sites coupled with the transfer to living plant host cells [6].

Many studies have been performed to predict the effect of quantitative and genetic bottlenecks on the overall fitness of plant virus quasispecies [7,8,9,10]. Yet, hardly any study addressed transmission bottlenecks with viruses for which nematodes are vectors. Only one study reported a bottleneck in the form of clearance of defective interfering RNAs from tobacco rattle tobravirus (TRV) populations as a result of transmission by the vector *Paratrichodorus pachydermus* [11].

Grapevine fanleaf virus (GFLV) is one of the most-studied nematode transmitted viruses due to its negative economic impact on grapevine (*Vitis* spp.), one of the oldest domesticated crops [12]. Over 70 viruses and five viroids have been identified so far as infecting grapevine with some agents considered as commensal viruses, while others cause extensive damages and are of great economic importance [13]. GFLV induces one the most severe viral disease of grapevine: the fanleaf degeneration disease which is responsible for serious losses in production [14,15,16]. The GFLV genome consists of two single-stranded positive-sense RNA molecules (RNA-1 and RNA-2). The genomic RNAs are encapsidated in icosahedral particles of about 30 nm in diameter, assembled from 60 units of the capsid protein in a pseudo T=3 symmetry [17]. RNA-1 and RNA-2 are necessary for local and systemic infection *in planta*. Both RNAs can be found in some particles as it was observed in purified preparations of GFLV [18]. While long distance dissemination of the virus results from the exchange of infected grapevine material, the virus is exclusively transmitted in the vineyard by the ectoparasitic dagger nematode *Xiphinema index* [19]. This nematode belongs to the *Longidoridae* family and is one of the 13 nematode species described as vectors of grapevine soil-borne viruses [20]. *Xiphinema index* acquires and releases GFLV particles during the feeding process when its stylet is deeply inserted in the parenchyma tissues of growing root tips. GFLV can be retained several years by *X. index,* even after extended periods of fallow beyond 4 years [21]. The transmission of GFLV by *X. index* occurs in a non-circulative and non-propagative manner [22]. While juveniles and adult can acquire and transmit the virus, GFLV is shed at each juvenile molting stages. The presence of the virus in the alimentary tract of the nematode does not affect traits of the life cycle of the vector such as its feeding behavior, reproduction or survival [23]. By analogy with other stylet-borne viruses, transmission of GFLV could result from highly specific interactions between the virion and a so far unknown nematode receptor. The determinant of transmission of GFLV corresponds to a domain exposed at the surface of the virion [24,25,26].

GFLV displays a high genetic diversity with up to 20% of nucleotide variability [27,28] and high genetic plasticity highlighted by the detection of many intra- and inter-species recombination events [29,30,31]. In addition, with the dawn of high throughput sequencing (HTS), mixed infections by many variants have been detected: at least two molecules of RNA-1 were detected in 10 out of 13 isolates from Champagne vineyards in France and remarkably five vines were co-infected by two or three RNA-1 and two RNA-2 molecules [32]. These mixed infections might result either from multiple sequential transmission events of single GFLV variants or from a single transmission event of different GFLV variants as noticed for other pathosystems [33]. The main objective of this study was to decipher the genetic composition of GFLV variants retained in a single *X. index* following acquisition from naturally infected grapevines and to identify a putative genetic bottleneck imposed by *X. index* on GFLV populations during the acquisition/retention phase of the transmission process. The complete genetic diversity of GFLV found by RNAseq in grapevines was not fully detected in nematodes. However, we observed that the combination of variants within a single nematode was different from that in individual plants, suggesting that nematodes could feed, acquire and withhold virus variants from multiple plants. This work will provide invaluable information on virus-nematode relationship as well as on GFLV epidemiology.

## 2. Materials and Methods

### 2.1. Grapevines and Nematode Rearings

All experiments were conducted in greenhouse located at the INRA-Grand Est Experimental Unit in Colmar, France (48.064457 lat., 7.334899 long.). Three GFLV-infected grapevines (*Vitis vinifera* cv. Chardonnay) named VA6, VA7 and VA8 derived from cuttings of about 20 years old vines (vine#4 in row#3, vine#1 in row#29 and vine#1 in row#30) in a fanleaf diseased vineyard in Chablis, France (47.80658 lat., 3.77889 long.). Cuttings were collected in July 2016 and propagated under greenhouse conditions. Plants were grown in small pots before their transplantation in a 10 L container in the presence of GFLV-free nematodes. Aviruliferous *X. index* were reared on fig plants (*Ficus carica*) as previously described [34]. An estimated population of 3700 aviruliferous *X. index* were allowed to feed on the three aforementioned grapevines starting in September 2016. Prior to setting up the feeding process, 5–6 leaves from each GFLV-infected grapevine were sampled, flash frozen in liquid nitrogen and stored at −80 °C for downstream high throughput sequencing (HTS) analyses. After 19 months (April 2018), nematodes were extracted from soil samples using the sieving method as previously described [35]. Adult *X. index* were individually handpicked under a binocular. Single nematodes (annotated i1 to i20) and pools of 30 individuals (annotated P1 to P5) were stored at −20 °C in sterile water prior to molecular analyses.

### 2.2. Total RNA Extraction from Grapevine Leaves, Roots Samples and Nematodes

Total RNA was extracted from 100 mg of grapevine tissue using the RNeasy Plant mini kit (Qiagen, Venlo, Netherlands), as previously described [32]. Purity and quality were assessed with a Bioanalyzer (Agilent, Santa Clara, CA, USA) prior to HTS analyses.

For *X. index*, total RNA extractions were performed as described previously [35] from nematode pools P1 to P5 and single nematodes i1 to i20.

### 2.3. High Throughput Sequencing (HTS), Bioinformatics and Phylogenetic Analyses

cDNA libraries were prepared from total RNA grape leaf extracts after a poly-A selection at the GeT-PlaGe Genotoul platform facility (INRA-Toulouse, France). The HTS approach was a paired-end 2 × 150 pb RNAseq completed on a Hiseq 3000 (Illumina, San Diego, CA, USA) following the manufacturer’s instructions. A double indexing strategy was used for all samples. After the demultiplexing steps being performed by GeT-PlaGe Genotoul, all dataset analyses were finalized using the CLC Genomics Workbench v11.0 software (Qiagen Bioinformatics, Aarhus, Denmark).

The virome of each grapevine sample was obtained by (i) mapping reads onto a curated list of grapevine-infecting virus references, and (ii) performing *de novo* assembly followed by BLAST [36] analyses, as previously described [37]. Complete to near-complete consensus genomes were obtained after extension of contigs by successive rounds of residual reads mapping.

All GFLV nucleotide sequences (GenBank numbers MN496417 to MN496426) were aligned manually using CLC workbench v11.0 with basic parameters (gap cost = 5, gap extension cost = 1). Phylogenetic trees were reconstructed using the Neighbor Joining method with nucleotide distance measures following Jukes-Cantor model. Bootstrapping analysis was performed with 1000 replicates. Phylogenetic trees were visualized using ITOL [38].

### 2.4. RT-PCR for Virus Detection

For grapevine leaves, roots and for nematodes’ pools, 20 ng aliquots of the total RNA as quantified by Nanodrop™ were used for RT-PCR whereas for single nematodes the whole total RNA extraction was used due to the low yield of RNA extraction. Firstly, the cDNA was obtained by using Superscript III reverse transcriptase (Invitrogen, Carlsbad, USA) with a mix of oligo(dT) in a final volume of 40 µL. Then, PCR was performed using 2 µL of the cDNA template using GoTaq^®^ DNA polymerase PCR kit (Promega, Madison, WI, USA), in a final volume of 50 µL as per the manufacturer’s recommendations. Degenerated primers targeting specifically GFLV RNA-1 were designed (Table S1) to amplify part of the RNA-dependent RNA polymerase (Pol) coding region located in the 3′-end of RNA-1 while those targeting GFLV RNA-2 were previously described for the amplification of part of the coat protein (CP) coding region located in the 3′-end of the RNA-2 [32]. The PCR cycling parameters were as follows: an initial denaturation at 95 °C for 2 min, followed by 38 cycles of 30 s at 95 °C, 30 s at 52 °C and 45 s at 72 °C, with a final 10 min at 72 °C. The PCR-amplified products were named amplicon 1 (RNA-1) and amplicon 2 (RNA-2). Five µL were resolved by electrophoresis in a 1.2% agarose gel in a 0.5× Tris Boric acid EDTA buffer and stained with ethidium bromide for observation of the 733 bp and 567 bp fragments in size for amplicon 1 and amplicon 2, respectively.

In parallel, PCR were performed on the same cDNAs to control the quality of the RNAs by using primer pairs designed to amplify part of the *X. index* actin (ACT) mRNA and *Vitis vinifera* glyceraldehyde 3-phosphate dehydrogenase (GAPDH) mRNA (Table S1). The list of primers designed to detect others virus species present in the source plants is also provided (Table S1).

### 2.5. RT-qPCR

To further investigate the accumulation of viruses in nematodes, RT-qPCR was performed using the same aforementioned cDNA. Eva Green qPCR mix (Biorad, Hercules, CA, USA) was used with specific primers (Table S1) targeting GFLV RNA-1, arabis mosaic virus (ArMV) RNA-2, grapevine rupestris vein feathering virus (GRVFV) and grapevine virus B (GVB) for real time amplification (Biorad CFX96^TM^ real-time system). Briefly, PCR was performed in 96-well optical reaction plates for 30 s at 95 °C followed by 40 cycles of denaturation for 5 s at 95 °C, annealing and elongation for 5 s at 65 °C. After each run, a melting curve analysis was carried out to ascertain that a single amplicon was produced in each well. Each sample/primer combination was carried out in triplicate from which average threshold cycle (Ct) values were obtained and used for calculation. Samples were considered positive only when all three biological replicates exhibited (i) a Ct value, (ii) a Standard Error (SE) of the triplicates below 0.5 and (iii) a Ct value + SE below Ct – SE of the negative control. Absolute quantification of GFLV RNA-1 was expressed as molecule of viral RNA per ng of total RNA and deduced from a linear regression of Ct values obtained from a serial dilution of plasmid containing cDNA from GFLV RNA-1 [32]. RT-qPCR data were also analyzed by calculating ΔCt that corresponds to the Ct value of the gene of interest minus the Ct value of the internal control (ACT for nematode and GAPDH for grapevine). ΔCt is inversely proportional to the quantity of the gene of interest (i.e., viral quantity). Relative quantification was performed based on the ΔΔCt method comparing other viruses to GFLV within each sample, or to the sample displaying the lowest quantity in the case of GFLV.

### 2.6. Restriction Fragment Length Polymorphism (RFLP)

RT-PCR amplicons 1 and 2 corresponding to fragments of cDNA of GFLV RNA-1 and RNA-2 respectively, were separately digested overnight at 37 °C. Digestions were performed with *Ava*I/*Ava*II or *Eco*RI (amplicon 1) or *Sty*I (amplicon 2) with 8 µL of PCR reaction from grapevine and pools of nematodes samples and 12 µL of PCR reaction from single nematodes samples in a final volume of 25 µL. Restriction enzymes were from New England Biolabs, Ipswich, MA, USA. Restriction digests were resolved by electrophoresis on 11% polyacrylamide (37.5:1) gels at 120 V for 90 min.

### 2.7. Cloning and Sanger Sequencing

The RT-PCR products of GFLV RNA-1 and RNA-2 from selected pools of nematodes and single nematodes were cloned into pGEM-T Easy Vector (Promega), as per the manufacturer’s recommendation, after a PCR cleanup step using the Nucleospin^®^ gel and PCR cleanup kit (Macherey-Nagel, Düren, Germany). Thirteen to forty-two clones per PCR products were Sanger sequenced (Genoscreen, Lille, France) using M13 universal primers.

## 3. Results

### 3.1. Sanitary Status of Grapevine Material and Identification of Multiple Variants of GFLV by HTS

The virome of the three grapevines VA6, VA7 and VA8 that were used as GFLV source plants in this study was determined by RNAseq. First, the dataset was analyzed for the presence of variants of GFLV. Complete to near-complete genomes of GFLV (i.e., covering at least the open reading frame [ORF] of both RNA-1 and RNA-2) were obtained in all three plants following *de novo* assembly analyses. From the three datasets, six GFLV-RNA1 and four GFLV RNA-2 consensus molecules were assembled. Each RNA molecule was identified in the text with the following code: the name of the sample, the number of the GFLV genomic RNA and the number of the molecular variant (Table 1). These sequences were submitted to GenBank (numbers MN496417 to MN496426). All RNA-1 and RNA-2 molecules segregated into 4 and 3 genetically distinct clades, respectively (Table 1 and Figure S1). The clades were arbitrarily named Ia to Id for RNA-1 and IIa to IIc for RNA-2 with an inter-clade nucleotide distance of at least 8%. None of these GFLV sequences were phylogenetically related to available sequences on GenBank (Figure S1). The range of nucleotide identity between sequences of the present work was 87.52–98.98% and 87.82–99.02% for the ORF1 and ORF2, respectively (Figure S1). The number of genetically distinct RNA-1 and -2 molecules differed for each plant. VA6 was the most complex vine with two molecules of each genomic RNA belonging to two different clades. The composition of GFLV variants for VA7 and VA8 (for which the mother-plants were adjacent in the Chablis vineyard) was similar but not identical. Both displayed the same RNA-2 variant (≈99% identity along the sequence) belonging to clade IIa as well as one RNA-1 molecule belonging to clade Ia. However, the second RNA-1 molecule belonged to clade Ib for VA7 and to clade Ia for VA8 (Table 1). Consequently, each plant was unique in its GFLV composition despite the fact that the three grapevines originated from the same vineyard.

The presence of viruses other than GFLV was further assessed in the three grapevines by analyzing the same RNAseq dataset. This was done by directly mapping total cleaned reads onto a curated collection of grapevine-infecting viruses’ reference sequences as previously described [37]. For the three grapevines, multiple variants of grapevine rupestris stem pitting-associated virus (GRSPaV), grapevine fleck virus (GFkV), and viroids (grapevine yellow speckle viroid-1, GYSVd-1 and hop stunt viroid, HSVd) were detected (Table 1). These viruses and viroids are commonly found in grapevines worldwide [39] and may be considered as part of the ‘background’ virome of grapevine. Other viruses belonging to the *Tymoviridae* family were also identified with grapevine redglobe virus (GRGV) in VA7 and with three molecular variants of grapevine rupestris vein feathering virus (GRVFV) in VA6 (Table 1). Arabis mosaic virus (ArMV), another nepovirus, was detected only in VA6. The same variant (≈99% identity along the genome) of grapevine virus B (GVB) was found in VA7 and VA8. Taken together, all plants were infected with at least three other virus species and two viroids, in addition to GFLV, confirming a high level of mixed infection in natural vineyard settings.

### 3.2. Distribution of GFLV Variants in Grapevine Plants by RT-PCR-RFLP

#### 3.2.1. Validation by RT-PCR-RFLP of Mixed Infections by GFLV Variants in Grapevines VA6, VA7 and VA8

**Figure 1:**
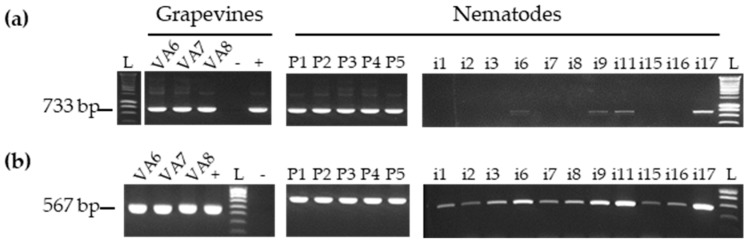
Specific detection of grapevine fanleaf virus (GFLV) RNA-1 (**a**) and RNA-2 (**b**) by RT-PCR in grapevines and nematodes (P: Pool of 30 nematodes and i: single nematode). L corresponds to the ladder, and + and - to positive and negative control respectively. The size of each product is indicated in base pairs (bp) on the left side of each gel.

Amplicons of the expected length for RNA-1 and RNA-2 were obtained by RT-PCR for all grapevine samples using the same total RNA extracts as for HTS (Figure 1, left panel).

**Figure 2:**
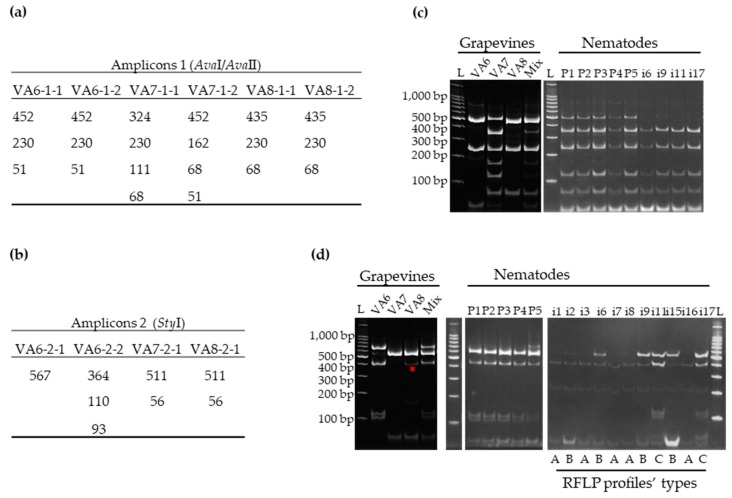
GFLV variants composition determined by restriction fragment length polymorphism (RFLP) on RT-PCR amplicons specific to RNA-1 and RNA-2, from grapevines VA6, VA7, and VA8 and from nematodes that fed on these vines (P: Pool of 30 nematodes and i: single nematodes). The theoretical length (in bp) of the different fragments generated by restriction digestion for amplicon 1 (**a**) and amplicon 2 (**b**) was determined for each molecular variant of GFLV *de novo* assembled sequence. RFLP profiles observed for amplicons 1 (**c**) and 2 (**d**). The size in base pairs (bp) of 100 bp ladder from Promega, is indicated on the left side of each gel. Mix corresponds to an artificial sample containing equimolar amounts of total RNA extracts from VA6, VA7, and VA8. The non-expected fragment for VA8 amplicon 2 is shown (*). Three different types of RFLP profiles (A, B and C) observed for amplicon 2 for single nematodes are indicated below the panel.

Restriction digestions of amplicons 1 and 2 with *Ava*I/*Ava*II and *Sty*I respectively, produced specific electrophoretic profiles for each sample (Figure 2c,d, left panel) that were consistent with the deduced in silico patterns (Figure 2a,b). Each grapevine displayed clear and distinct RNA-1 profiles, while RFLP patterns for VA7 and VA8 RNA-2 were indistinguishable since both grapevines were infected with the same variants from clade IIa (Figure S1). Mixing the total RNA extracts obtained from plants VA6, VA7 and VA8 prior to performing the RT-PCR-RFLP gave the correct and complete profile with all the expected fragments. For VA8 amplicon 2, a non-expected fragment of 300–400 bp size was observed (Figure 2d, left panel*). Indeed, when reanalyzing the HTS data, we were able to observe a SNP (Single Nucleotide Polymorphism) at position 144 of the RNA-2 amplicon mutating the initial C into T at a rate of 18.33% (out of 10 454 reads covering that particular nucleotide), thus generating an additional *Sty*I restriction site. These results indicated that RT-PCR-RFLP corroborated perfectly the GFLV diversity detected by HTS.

#### 3.2.2. Distribution of GFLV Variants in Different Organs of Two VA6 Grapevine Cuttings

**Figure 3:**
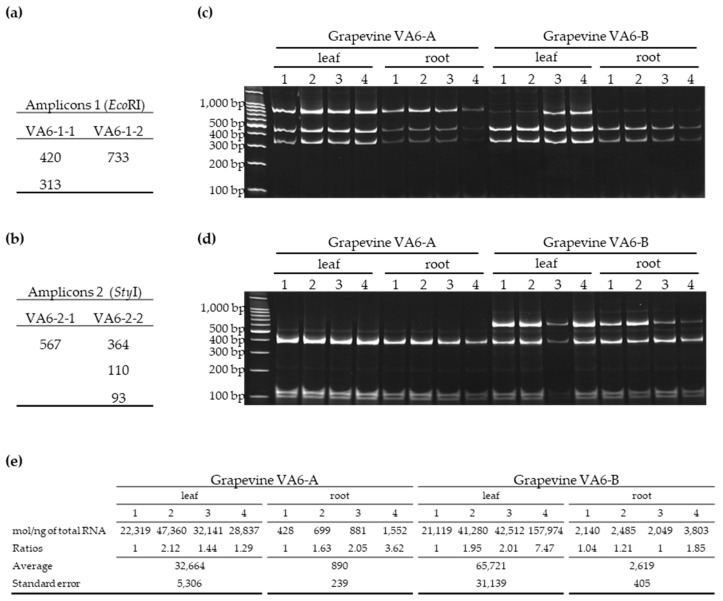
Comparison of RFLP profiles and viral load of eight different leaf and root samples from two grapevine cuttings (VA6-A and VA6-B). The theoretical length of different fragments generated by restriction digestion for amplicon 1 (**a**) and amplicon 2 (**b**) was determined for each molecular variant of GFLV *de novo* assembled from grapevine VA6. RFLP profiles observed for amplicons 1 (**c**) and 2 (**d**). The size in base pairs (bp) is indicated on the left side of each gel. The viral load (e) estimated by absolute quantification of RNA-1 by RT-qPCR was expressed in molecules/ng of total RNA as previously described [32]. The ratios between samples from a same tissue and a same plant were calculated using as the denominator the sample with the lowest viral amount. Average and standard error were calculated within a compartment (leaf or root) per plant, allowing for direct comparison between organs and plants.

The distribution of GFLV variants within a grapevine, was determined in grapevine VA6 since it is the only plant infected with two genetically distinct molecules of both RNA-1 and RNA-2 (Table 1). Cuttings generated from the mother stock vine VA6 were obtained in September 2016 and cultivated in pots in the absence of nematodes and the two resulting plants named VA6-A and VA6-B were grown in a greenhouse. Canes were pruned in the winter and a shoot of 1.80 m was allowed to grow in the following spring. Four random samples from the canopy and four random samples from the root system of VA6-A and VA6-B were collected in May 2019 for total RNA extraction. All samples were positive for the presence of GFLV RNA-1 and RNA-2 molecules by specific RT-PCR (Figure 3c,d). Analyses of GFLV variants by RFLP after digestion with *Eco*RI and *Sty*I of RNA-1 and RNA-2 amplicons, respectively, showed the presence of all molecular variants as expected by in silico analyses (Figure 3). Both grapevines displayed similar profile patterns for amplicon 1. Although RFLP is only a semi-quantitative technique, differences in variants accumulation were observed, not only between plants but also within a given vine and for different organs (see grapevine VA6-B, leaf 1 and 2 versus leaf 3 and 4 and root 1, 2, 3, 4 in Figure 3). The 733 bp fragment was hardly visible likely due to a small amount of molecule VA6-1-2. RNA-2 profiles were different between plants, with the 567 bp band (corresponding to VA6-2-1 molecule) being clearly visible in grapevine VA6-B but absent in VA6-A.

The GFLV accumulation, estimated by absolute RT-qPCR, in the same 16 cDNA samples from plants VA6-A and VA6-B, indicated a relative uniform quantity of RNA-1 within organs, such as roots and leaves of a same plant, with small ratios between samples comprised between 1 and 2.12 except for 2 samples with ratios of 7.47 and 3.62 (Figure 3e). However, viral load in roots was between 25 times and 36 times lower than in leaves.

### 3.3. Detection of GFLV Variants in Pooled and Single Nematodes

In order to determine the genetic composition of GFLV populations in nematodes, we first assessed the RNA quality of total RNA extracts obtained for the five pools of 30 nematodes and 20 single nematodes. All five pools were positive for *X. index* mRNA actin (ACT) but only 11 out of 20 single nematodes tested positive for ACT (Figure S2). Therefore, all downstream analyses were then performed only on the 11 ACT-positive single nematodes and on pools P1-P5. To ascertain that detection of GFLV and/or other viruses was specific to nematodes, potential contamination by grapevine impurities was assessed by RT-PCR using *V. vinifera* glyceraldehyde 3-phosphate dehydrogenase (GAPDH) primers (Table S1). No amplification of GAPDH was detected in any of the pooled or single nematode extracts by RT-PCR (Figure S2).

The presence of GFLV in nematodes was first evaluated by RT-PCR using the same primers as described above (Table S1). GFLV RNA-1 and RNA-2 were readily detected in the five pools of 30 nematodes (Figure 1, right panel). The presence of RNA-2 was also observed in all 11 single nematodes tested. However, GFLV RNA-1 was only detected in four single nematodes (i6, i9, i11, and i17). The lack of detection of RNA-1 compared to RNA-2 in most single nematodes could be due to RNA-1 quantities below the detection threshold level of RT-PCR as already noticed in our previous work [32]. Indeed, the use of RT-qPCR, a more sensitive technique compared to RT-PCR, allowed the detection of GFLV-RNA1 in 9 out of 11 nematodes tested (Table S2).

The GFLV populations within pooled and single nematodes were assessed by RFLP (Figure 2c,d, right panel). RNA-1 restriction digestion profiles from pools (P) showed more fragments than those from single (i) nematodes. They were also more complex than profiles obtained from single grapevine VA6, VA7 or VA8 but not as complex as the profile from the artificial mix of the three grapevines (Figure 2). While the RFLP profiles obtained for amplicons 2 were similar for the five pools of nematodes, they were different for single nematodes with at least three categories of profiles (Figure 2c,d, right panel).

**Figure 4:**
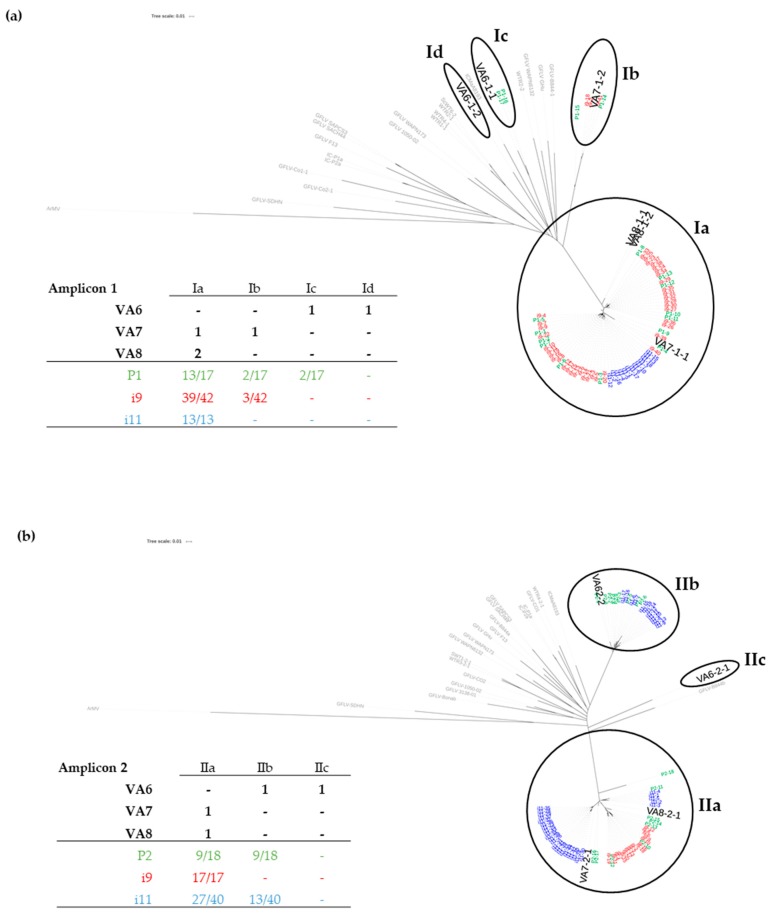
Phylogenetic trees based on the Neighbor Joining method after alignment of sequences corresponding to amplicon 1 (**a**) and amplicon 2 (**b**). The sequences obtained from infected plants are in grey for the known GFLV sequences retrieved from GenBank and in bold for those from our three grapevine sources (VA6, VA7, and VA8) as deduced from our RNAseq analyses. The sequences acquired from nematodes and obtained by Sanger sequencing of clones of amplicons are given in green (pool of 30 nematodes P1 and P2), in red (single nematode i9) and in blue (single nematode i11).The tables summarize the distribution of GFLV molecular variants and clones in each clade. The composition of molecular GFLV variants deduced from RNAseq for each plant is represented in black. For nematodes, the same color code as above (green, red and blue) is used and the ratios of clones belonging to a clade (over the total number of clones sequenced for each sample) are given.

To further determine GFLV population in nematodes, cloning and Sanger-sequencing were performed from P1 and P2 nematode pools for RNA1 and RNA2, respectively. Phylogenetic trees showed that nearly the whole GFLV diversity was detected in these pools of nematodes with 17 and 18 clones sequenced for RNA-1 and RNA-2, respectively (Figure 4). For example, three clades out of the four that constituted the RNA-1 diversity were identified in P1. Only one variant, VA6-1-2, was not detected. Regarding RNA-2 in the P2 pool, only VA6-2-1 was not detected. These variants were also not detected by RFLP in some samples from leaf and root of the VA6-A and VA6-B plants, which could potentially allow less accumulation of these molecules in roots on which nematodes were feeding. This would explain the absence of their detection in nematodes.

Slightly different results were obtained for single nematodes (i9 and i11) where RNA-1 molecular variants from only two clades (Ia and Ib) were detected. Regarding RNA-2, results were similar to those of pooled nematodes with variants from clades IIa and IIb being detected in single nematode i11. As molecular variants from these clades were present in different plants, these results revealed a complex GFLV RNA-2 diversity within a single nematode deriving from at least two different grapevines, VA6 and VA7 and/or VA8.

### 3.4. Detection of Other Viruses in Pool and Single Nematodes

As viruses other than GFLV were detected in the three grapevines source plants, their presence in nematodes was also assessed. No vector has been identified so far for GRVFV and GRSPaV from the *Tymoviridae* and *Betaflexiviridae* families, respectively. ArMV is specifically transmitted by the dagger nematode *X. diversicaudatum* while mealybugs and soft scale insects transmit GVB from the *Vitivirus* genus [13]. Pools of *X. index* and single *X. index* were examined by RT-PCR (Figure S3) and RT-qPCR (Table S2). Surprisingly, GRVFV was detected in four out of the five nematode pools by RT-PCR, while ArMV that was detected in the same plant as GRVFV (VA6), was not detected in nematode pools. Also, GRSPaV and GVB were not detected in the pools of nematodes by RT-PCR (Figure S3). In two selected single nematodes, i9 and i11, no virus other than GFLV was detected (Figure S3). Overall, these results were consolidated by RT-qPCR (Table S2), a more sensitive technique, which improved only the detection of ArMV for 2 out of the 5 pools. The constant detection of GFLV in nematodes was striking compared to the other viruses stressing the exclusive relationship and solid interaction between GFLV and its nematode vector.

## 4. Discussion

Concomitant with the use of HTS, it is quite the rule to find grapevine being mixed-infected with many different viruses [32,37,39,40,41,42,43]. Here, once again, we report a complex virome in each of the three grapevines collected from a fanleaf-diseased vineyard in Chablis, France (Table 1). These vines were infected not only with GFLV but also with different combinations of six other viruses belonging to several viral families and two viroids. In addition to the grapevine commensal ‘background’ viruses and viroids (i.e., GRSPaV, GFkV, HSVd, GYSVd), ArMV, GVB, GRVFV, and GRGV were detected by RNAseq. This high level of viral association may play an important role for viruses’ evolution in grapevine. It may also provide opportunities for the exchange of genetic material via intra- and inter-species recombinations [29,30,31] and/or the reassortment of genomes from the same species or closely related virus species. The latter event has yet to be observed in grapevine, unlike in other pathosystems [44,45].

The co-infections status in grapevines VA6, VA7 and VA8 were not only remarkable when looking at the genus/family levels; it was also meaningful when focusing on a single species of virus such as GFLV. A *de novo* assembly analysis following RNAseq confirmed previous observations on the co-existence of multiple genetic variants of GFLV in a single grapevine [29,32]. As already observed, more genetically different RNA-1 molecules were detected in the three grapevines compared to RNA-2, with six distinct GFLV RNA-1 and four RNA-2 molecules assembled from RNAseq data. These molecules exhibited up to 12% of nucleotide divergence along the ORF-coding sequences (Figure S1). Most of them belonged to different phylogenetic clades and were distinguishable by RT-PCR-RFLP. GFLV RNA-2/RNA-1 RPKM ratios (Table 1) were consistent with previous findings [29,32], confirming that RNA-2 molecules are always present in a greater amount than RNA-1 molecules within a sample. Interestingly, the distribution of the complex genetic diversity of GFLV found in VA6 (two RNA-1 and two RNA-2 molecules belonging to distinct clades) was fairly uniform within the grapevine in leaves and roots (Figure 3), suggesting no significant tissue tropism with segregation of GFLV haplotypes between different organs of the same plant. However, differences in variant ratios were clearly visible when two cuttings (VA6-A and -B) isolated from the same mother plant were tested by RFLP. This indicated a heterogeneous distribution of the GFLV variants within a plant, pinpointing a possible stochastic genetic bottleneck [46]. In addition, a quantitative bottleneck was clearly observed between the two organs of the plant tested, with viral molecules being accumulated about 30 times less in roots than in leaves. To confirm these findings, additional work needs to be performed by exploring the spatio-temporal distribution of GFLV variants within single grapevine plants [47].

Many theoretical studies have been performed to predict the effect of population and genetic bottlenecks on the overall plant-viral fitness [7,8,9,10]. The size of bottlenecks during horizontal transmission has been estimated in a few plant pathosystems, mostly involving aphid vectors [48,49,50], reflecting the fact that insects are the largest class of plant virus-transmitting vectors [51]. For example, indirect evidence of narrow bottlenecks during transmission were provided by series of mechanical inoculation of potato virus Y (PVY) that led to the emergence of poorly aphid-transmitted variants, otherwise selected out during the natural insect-mediated inoculation [52]. In addition, a severe genetic bottleneck imposed during transmission by two aphid species was observed under laboratory conditions by using an artificial population of 14 cucumber mosaic virus (CMV) mutants in squash [5]. Similar observations were drawn from studies involving citrus tristeza virus (CTV) under field conditions [53,54]. Comparable studies were also performed on animal-viruses [55,56,57,58,59]. To date, little is known about bottlenecks imposed on virus populations during transmission by nematodes either at the acquisition/retention phase, or during the inoculation phase. In the present work, we focused in detecting GFLV post-acquisition in its specific vector, *X. index*.

Here, this is the first report showing the presence of both RNA-1 and RNA-2 molecules of GFLV in most single nematodes tested following a 19-months feeding period on infected grapevines. All single nematodes were positive for GFLV by using PCR technics targeting RNA-2 molecules (11/11) and more than 80% when focusing on RNA-1 (Figure 1, Table S2). This result is important, suggesting that, as often assumed but never demonstrated, a single nematode could be at the origin of an infection by introducing into a grapevine cell both GFLV-RNAs which are required for the virus to complete its viral cycle. Other viruses present in the plants (e.g., ArMV and GRVFV) were also detected in nematodes, however, never reaching such high frequency as GFLV, suggesting that their presence in the vector might only be the results of the vector feeding onto the root system and not be due to a specific virus acquisition/retention and interaction *per se*.

In addition, we demonstrated that a single nematode could acquire and retain different variants of GFLV after feeding not only on one, but possibly on two different plants. For example, it is clear that single nematode #11 carried RNA-2 variants derived from at least 2 vines, VA6, VA7 and/or VA8 (Figure 4b). This can be due to (i) feeding and acquiring sequentially the variants from the two plants or (ii) acquiring variants at once as the result of a superinfection that had happened during the time lapse of the assay. The latter option was ruled out after all three grapevines tested negative for superinfection post-infestation by nematodes using RT-PCR-RFLP on total RNA extracts obtained from leaves of the vines. By retaining multiple molecules of GFLV RNAs coming from different vines, *X. index* could enhance the number of possible combinations between RNA-1 and RNA-2 molecules that it might deliver during the inoculation phase of the transmission process. This could increase potential reassortments, one of the main driving forces of virus evolution, among mutation and recombination events.

Despite the limited number of nematodes being tested, the genetic diversity of GFLV from the three grapevines was almost but not completely recovered in nematodes, with RNA molecules VA6-1-2 and VA6-2-1 not detected in nematodes. This partial genetic bottleneck could be explained by: (i) a non-uniform distribution of populations of GFLV within the plant VA6 (molecules VA6-1-2 and VA6-2-1 were hardly detected by RFLP in some samples from roots of grapevine cuttings VA6-A and VA6-B), and (ii) as previously mentioned [60], cloning and Sanger-sequencing might not be the best method (not resolutive enough, unlike HTS) to uncover the complete genetic diversity of a viral population.

To date, we do not know whether all the GFLV variants carried by viruliferous *X. index* are transferred into plants during inoculation and can be detected in bait grapevines. Yet, if our results can be transferred to the field, the spread of GFLV within a vineyard will tend to be maintained in the long term, due to the capacity of a single nematode to retain multiple molecular variants. These findings are fundamental concerning GFLV evolution and for the successful establishment of a robust systemic infection in grapevine regardless of challenges encountered in distinct organs and under different environmental pressure.

## 5. Conclusion

In this work, we report that the large diversity and genetic composition of GFLV populations found in grapevines was maintained quite well in a population of nematodes as well as in single nematodes. This might reflect the putative long co-evolution between GFLV and its nematode vector [61], for which grapevine is the main and probably only host. This tight association between the three partners may be explained by (i) the mode of culture of grapevine, (ii) the mode of transmission of the virus and (iii) the biology of the vector. First, the monoculture of this perennial plant in vineyards terroirs maintains population of *X. index*, ultimately improving their efficiency to survive and disseminate the virus. Second, concerning the mode of transmission, and as previously stated [60], it is possible that the ‘helper strategy’ transmission may be less prone to severe bottlenecks than the ‘capsid strategy’ transmission where virions interact directly with the vector as suspected for GFLV. Our results suggesting a large bottleneck on GFLV populations could indirectly help hypothesize on the so-far unknown mode of transmission by *X. index*. Third, unlike insect vectors, nematodes behave more like colonizers than migrators, implying a slow but inexorable spread of an infection within a vineyard, and that the viral genetic diversity, if needed to be conserved, must be assured by transmission with a limited number of vectors. In addition, it is interesting to notice that within vineyards, grapevine is often mixed-infected with multiple GFLV variants [27,32], this work. This could be due to either multiple successive infections by single-variant-carrying viruliferous nematodes or a single infection by nematodes carrying different variants. This present study suggests the latter.

## Figures and Tables

**Table 1:** Virome of three grapevines with their ID and the total number of clean reads obtained from RNAseq data. Abbreviations of viruses and viroids names found in the three grapevines are given. The presence of each viral variants *de novo* assembled, for which 100% of the genome or at least all ORFs are covered, is expressed in RPKM (Reads per Kilobase per Million reads). The presence of other viruses, for which the genome could not be assembled or not fully covered, is shown by a ✓. Parameters were set at 0.7 for length and 0.8 for identity using CLC workbench. The name of each GFLV variants and the clade to which they belong are given in parenthesis and italics. For viruses other than GFLV, if more than one variant was identified, its number is indicated after the abbreviation of the virus and the viroid names.

	ID	VA6	VA7	VA8
	Total clean reads	32,435,733	30,338,401	34,907,736
**GFLV**	RNA1-1	772 *(VA6-1-1, clade Ic)*	680 *(VA7-1-1, clade Ia)*	670 *(VA8-1-1, clade Ia)*
	RNA1-2	235 *(VA6-1-2, clade Id)*	194 *(VA7-1-2, clade Ib)*	577 *(VA8-1-2, clade Ia)*
	RNA2-1	904 *(VA6-2-1, clade IIc)*	2037 *(VA7-2-1, clade IIa)*	2923 *(VA8-2-1, clade IIa)*
	RNA2-2	899 *(VA6-2-2, clade IIb)*	-	-
**ArMV**	RNA1	13	-	-
	RNA2	26	-	-
**Other viruses**	GVB	-	55	105
	GRVFV-1	15	-	-
	GRVFV-2	13	-	-
	GRVFV-3	11	-	-
	GRGV	-	✓	-
**“background” Virome (viruses And viroids)**	GRSPaV-1	37	41	20
	GRSPaV-2	16	28	19
	GRSPaV-3	3	26	11
	GRSPaV-4	-	23	10
	GRSPaV-5	-	7	5
	GFkV	✓	✓	✓
	GYSVd1-1	91	103	43
	GYSVd1-2	-	49	-
	HSVd	112	25	181

## Supplementary Materials

The following ones are available online at https://www.mdpi.com/1999-4915/11/12/1139/s1, Figure S1: Genetic diversity of GFLV sequences from grapevines VA6, VA7 and V8. Figure S2: Assessment by RT-PCR of mRNA quality from *X. index* nematodes. Figure S3: Detection by RT-PCR of viruses other than GFLV in nematodes. Table S1: List of primers. Table S2: Relative quantification by RT-qPCR of viruses other than GFLV in grapevines or nematodes.

## References

[1] Steinhauer D.A., Domingo E., Holland J.J. (1992). Lack of evidence for proofreading mechanisms associated with an RNA virus polymerase. Gene.

[2] Domingo E., Sheldon J., Perales C. (2012). Viral Quasispecies Evolution. Microbiol. Mol. Biol. Rev..

[3] Roossinck M.J. (1997). Mechanisms of plant virus evolution. Annu. Rev. Phytopathol..

[4] Li H., Roossinck M.J. (2004). Genetic Bottlenecks Reduce Population Variation in an Experimental RNA Virus Population. J. Virol..

[5] Ali A., Li H., Schneider W.L., Sherman D.J., Gray S., Smith D., Roossinck M.J. (2006). Analysis of genetic bottlenecks during horizontal transmission of Cucumber mosaic virus. J. Virol..

[6] Dader B., Then C., Berthelot E., Ducousso M., Ng J.C.K., Drucker M. (2017). Insect transmission of plant viruses: Multilayered interactions optimize viral propagation. Insect Sci..

[7] Abel S., Abel zur Wiesch P., Davis B.M., Waldor M.K. (2015). Analysis of Bottlenecks in Experimental Models of Infection. PLoS Pathog..

[8] Monsion B., Froissart R., Michalakis Y., Blanc S. (2008). Large Bottleneck Size in Cauliflower Mosaic Virus Populations during Host Plant Colonization. PLoS Pathog..

[9] Zwart M.P., Daròs J.-A., Elena S.F. (2011). One Is Enough: In Vivo Effective Population Size Is Dose-Dependent for a Plant RNA Virus. PLoS Pathog..

[10] Zwart M.P., Elena S.F. (2015). Matters of Size: Genetic Bottlenecks in Virus Infection and Their Potential Impact on Evolution. Annu. Rev. Virol..

[11] Visser P.B., Brown D.J.F., Brederode F.T., Bol J.F. (1999). Nematode Transmission of Tobacco Rattle Virus Serves as a Bottleneck to Clear the Virus Population from Defective Interfering RNAs. Virology.

[12] Reynolds A.G., Meng B., Martelli G.P., Golino D.A., Fuchs M. (2017). The Grapevine, Viticulture, and Winemaking: A Brief Introduction. Grapevine Viruses: Molecular Biology, Diagnostics and Management.

[13] Martelli G.P., Meng B., Martelli G.P., Golino D.A., Fuchs M. (2017). An Overview on Grapevine Viruses, Viroids, and the Diseases They Cause. Grapevine Viruses: Molecular Biology, Diagnostics and Management.

[14] Digiaro M., Elbeaino T., Martelli G.P., Meng B., Martelli G.P., Golino D.A., Fuchs M. (2017). Grapevine fanleaf virus and Other Old World Nepoviruses. Grapevine Viruses: Molecular Biology, Diagnostics and Management.

[15] Vigne E., Komar V., Tannières M., Demangeat G., Duchêne E., Steyer D., Lemarquis G., Ritzenthaler C., Lemaire O. Comparative pathogenic effects of distinct Grapevine fanleaf virus strains on *Vitis vinifera* cvs Gewurztraminer and Chardonnay. Proceedings of the 18th Congress of the International Council for the Study of Virus and Virus-Like Diseases of the Grapevine (ICVG).

[16] Andret-Link P., Laporte C., Valat L., Ritzenthaler C., Demangeat G., Vigne E., Laval V., Pfeiffer P., Stussi-Garaud C., Fuchs M. (2004). Grapevine fanleaf virus: Still a major threat to the grapevine industry. J. Plant Pathol..

[17] Schmitt-Keichinger C., Hemmer C., Berthold F., Ritzenthaler C., Meng B., Martelli G.P., Golino D.A., Fuchs M. (2017). Molecular, Cellular, and Structural Biology of Grapevine fanleaf virus. Grapevine Viruses: Molecular Biology, Diagnostics and Management.

[18] Quacquarelli A., Gallitelli D., Savino V., Martelli G.P. (1976). Properties of Grapevine Fanleaf Virus. J. Gen. Virol..

[19] Hewitt W.B., Raski D.J., Goheen A.C. (1958). Nematode vector of soil-borne fanleaf virus of grapevines. Phytopathology.

[20] Andret-Link P., Marmonier A., Belval L., Hleibieh K., Ritzenthaler C., Demangeat G., Meng B., Martelli G.P., Golino D.A., Fuchs M. (2017). Ectoparasitic Nematode Vectors of Grapevine Viruses. Grapevine Viruses: Molecular Biology, Diagnostics and Management.

[21] Demangeat G., Voisin R., Minot J.-C., Bosselut N., Fuchs M., Esmenjaud D. (2005). Survival of Xiphinema index in Vineyard Soil and Retention of Grapevine fanleaf virus Over Extended Time in the Absence of Host Plants. Phytopathology.

[22] Taylor C.E., Raski D.J. (1964). On the Transmission of Grape Fanleaf By Xiphinema Index. Nematologica.

[23] Das S., Raski D.J. (1969). Effect of Grapevine Fanleaf Virus on the Reproduction and Survival of its Nematode Vector, Xiphinema index Thorne &Allen. J. Nematol..

[24] Andret-Link P., Schmitt-Keichinger C., Demangeat G., Komar V., Fuchs M. (2004). The specific transmission of Grapevine fanleaf virus by its nematode vector Xiphinema index is solely determined by the viral coat protein. Virology.

[25] Schellenberger P., Andret-Link P., Schmitt-Keichinger C., Bergdoll M., Marmonier A., Vigne E., Lemaire O., Fuchs M., Demangeat G., Ritzenthaler C. (2010). A stretch of 11 amino acids in the betaB-betaC loop of the coat protein of grapevine fanleaf virus is essential for transmission by the nematode Xiphinema index. J. Virol..

[26] Schellenberger P., Sauter C., Lorber B., Bron P., Trapani S., Bergdoll M., Marmonier A., Schmitt-Keichinger C., Lemaire O., Demangeat G. (2011). Structural Insights into Viral Determinants of Nematode Mediated Grapevine fanleaf virus Transmission. PLoS Pathog..

[27] Vigne E., Bergdoll M., Guyader S., Fuchs M. (2004). Population structure and genetic variability within isolates of Grapevine fanleaf virus from a naturally infected vineyard in France: Evidence for mixed infection and recombination. J. Gen. Virol..

[28] Zhou J., Fan X., Dong Y., Zhang Z., Ren F., Hu G., Li Z. (2017). Complete nucleotide sequence of a new variant of grapevine fanleaf virus from northeastern China. Arch. Virol..

[29] Hily J.-M., Demanèche S., Poulicard N., Tannières M., Djennane S., Beuve M., Vigne E., Demangeat G., Komar V., Gertz C. (2018). Metagenomic-based impact study of transgenic grapevine rootstock on its associated virome and soil bacteriome. Plant Biotechnol. J..

[30] Vigne E., Demangeat G., Komar V., Fuchs M. (2005). Characterization of a naturally occurring recombinant isolate of Grapevine fanleaf virus. Arch. Virol..

[31] Vigne E., Marmonier A., Fuchs M. (2008). Multiple interspecies recombination events within RNA2 of Grapevine fanleaf virus and Arabis mosaic virus. Arch. Virol..

[32] Vigne E., Garcia S., Komar V., Lemaire O., Hily J.-M. (2018). Comparison of Serological and Molecular Methods With High-Throughput Sequencing for the Detection and Quantification of Grapevine Fanleaf Virus in Vineyard Samples. Front. Microbiol..

[33] Syller J. (2014). Biological and molecular events associated with simultaneous transmission of plant viruses by invertebrate and fungal vectors. Mol. Plant Pathol..

[34] Demangeat G., Komar V., Van-Ghelder C., Voisin R., Lemaire O., Esmenjaud D., Fuchs M. (2010). Transmission Competency of Single-Female Xiphinema index Lines for Grapevine fanleaf virus. Phytopathology.

[35] Demangeat G., Komar V., Cornuet P., Esmenjaud D., Fuchs M. (2004). Sensitive and reliable detection of grapevine fanleaf virus in a single Xiphinema index nematode vector. J. Virol. Methods.

[36] Altschul S.F., Gish W., Miller W., Myers E.W., Lipman D.J. (1990). Basic local alignment search tool. J. Mol. Biol..

[37] Hily J.-M., Candresse T., Garcia S., Vigne E., Tannière M., Komar V., Barnabé G., Alliaume A., Gilg S., Hommay G. (2018). High-Throughput Sequencing and the Viromic Study of Grapevine Leaves: From the Detection of Grapevine-Infecting Viruses to the Description of a New Environmental Tymovirales Member. Front. Microbiol..

[38] Letunic I., Bork P. (2016). Interactive tree of life (iTOL) v3: An online tool for the display and annotation of phylogenetic and other trees. Nucleic Acids Res..

[39] Czotter N., Molnar J., Szabó E., Demian E., Kontra L., Baksa I., Szittya G., Kocsis L., Deak T., Bisztray G. (2018). NGS of Virus-Derived Small RNAs as a Diagnostic Method Used to Determine Viromes of Hungarian Vineyards. Front. Microbiol..

[40] Beuve M., Hily J.M., Alliaume A., Reinbold C., Le Maguet J., Candresse T., Herrbach E., Lemaire O. (2018). A complex virome unveiled by deep sequencing analysis of RNAs from a French Pinot Noir grapevine exhibiting strong leafroll symptoms. Arch. Virol..

[41] Huogen X., Caihong L., Maher A.R., Valerian D., Baozhong M. (2019). Metagenomic Analysis of Riesling Grapevine Reveals a Complex Virome Including Two New and Divergent Variants of Grapevine leafroll-associated virus 3. Plant Dis..

[42] Jo Y., Choi H., Cho J.K., Yoon J.Y., Choi S.K., Cho W.K. (2015). In silico approach to reveal viral populations in grapevine cultivar Tannat using transcriptome data. Sci. Rep..

[43] Lunden S., Meng B., Avery J., Qiu W. (2009). Association of Grapevine fanleaf virus, Tomato ringspot virus and Grapevine rupestris stem pitting-associated virus with a grapevine vein-clearing complex on var. Chardonnay. Eur. J. Plant Pathol..

[44] Wright S.M., Kawaoka Y., Sharp G.B., Senne D.A., Webster R.G. (1992). Interspecies Transmission and Reassortment of Influenza a Viruses in Pigs and Turkeys in the United States. Am. J. Epidemiol..

[45] Schumann T., Hotzel H., Otto P., Johne R. (2009). Evidence of interspecies transmission and reassortment among avian group A rotaviruses. Virology.

[46] Gutierrez S., Michalakis Y., Blanc S. (2012). Virus population bottlenecks during within-host progression and host-to-host transmission. Curr. Opin. Virol..

[47] Krebelj A., Cepin U., Ravnikar M., Pompe Novak M. (2015). Spatio-temporal distribution of Grapevine fanleaf virus (GFLV) in grapevine. Eur. J. Plant Pathol..

[48] Betancourt M., Fereres A., Fraile A., García-Arenal F. (2008). Estimation of the effective number of founders that initiate an infection after aphid transmission of a multipartite plant virus. J. Virol..

[49] Moury B., Fabre F., Senoussi R. (2007). Estimation of the number of virus particles transmitted by an insect vector. Proc. Natl. Acad. Sci. USA.

[50] Gallet R., Fabre F., Thébaud G., Sofonea M.T., Sicard A., Blanc S., Michalakis Y. (2018). Small Bottleneck Size in a Highly Multipartite Virus during a Complete Infection Cycle. J. Virol..

[51] Bragard C., Caciagli P., Lemaire O., Lopez-Moya J.J., MacFarlane S., Peters D., Susi P., Torrance L. (2013). Status and Prospects of Plant Virus Control Through Interference with Vector Transmission. Annu. Rev. Phytopathol..

[52] Legavre T., Maia I.G., Casse-Delbart F., Bernardi F., Robaglia C. (1996). Switches in the mode of transmission select for or against a poorly aphid-transmissible strain of potato virus Y with reduced helper component and virus accumulation. J. Gen. Virol..

[53] Nolasco G., Fonseca F., Silva G. (2008). Occurrence of genetic bottlenecks during citrus tristeza virus acquisition by Toxoptera citricida under field conditions. Arch. Virol..

[54] Albiach-Martí M.R., Guerri J., de Mendoza A.H., Laigret F., Ballester-Olmos J.F., Moreno P. (2000). Aphid Transmission Alters the Genomic and Defective RNA Populations of Citrus tristeza virus Isolates. Phytopathology.

[55] Keele B.F., Giorgi E.E., Salazar-Gonzalez J.F., Decker J.M., Pham K.T., Salazar M.G., Sun C., Grayson T., Wang S., Li H. (2008). Identification and characterization of transmitted and early founder virus envelopes in primary HIV-1 infection. Proc. Natl. Acad. Sci. USA.

[56] Smith D.R., Adams A.P., Kenney J.L., Wang E., Weaver S.C. (2008). Venezuelan equine encephalitis virus in the mosquito vector Aedes taeniorhynchus: Infection initiated by a small number of susceptible epithelial cells and a population bottleneck. Virology.

[57] Forrester N.L., Guerbois M., Seymour R.L., Spratt H., Weaver S.C. (2012). Vector-borne transmission imposes a severe bottleneck on an RNA virus population. PLoS Pathog..

[58] Lequime S., Fontaine A., Ar Gouilh M., Moltini-Conclois I., Lambrechts L. (2016). Genetic Drift, Purifying Selection and Vector Genotype Shape Dengue Virus Intra-host Genetic Diversity in Mosquitoes. PLoS Genet..

[59] Grubaugh N.D., Weger-Lucarelli J., Murrieta R.A., Fauver J.R., Garcia-Luna S.M., Prasad A.N., Black W.C., Ebel G.D. (2016). Genetic Drift during Systemic Arbovirus Infection of Mosquito Vectors Leads to Decreased Relative Fitness during Host Switching. Cell Host Microbe.

[60] Simmons H.E., Dunham J.P., Stack J.C., Dickins B.J.A., Pagán I., Holmes E.C., Stephenson A.G. (2012). Deep sequencing reveals persistence of intra-and inter-host genetic diversity in natural and greenhouse populations of zucchini yellow mosaic virus. J. Gen. Virol..

[61] Nguyen V.C., Villate L., Gutierrez-Gutierrez C., Castillo P., Van Ghelder C., Plantard O., Esmenjaud D. (2019). Phylogeography of the soil-borne vector nematode Xiphinema index highly suggests Eastern origin and dissemination with domesticated grapevine. Sci. Rep..

