# Supplementary material for: Detection of Multiple Variants of Grapevine Fanleaf Virus in Single Xiphinema index Nematodes
Source: Viruses. 2019 Dec 10;11(12):1139. doi: 10.3390/v11121139 (PMC6950412; doi:10.3390/v11121139)
Supplement: Supplementary file 1 [file viruses-11-01139-s001.zip › FigureS1.pdf]

(a)

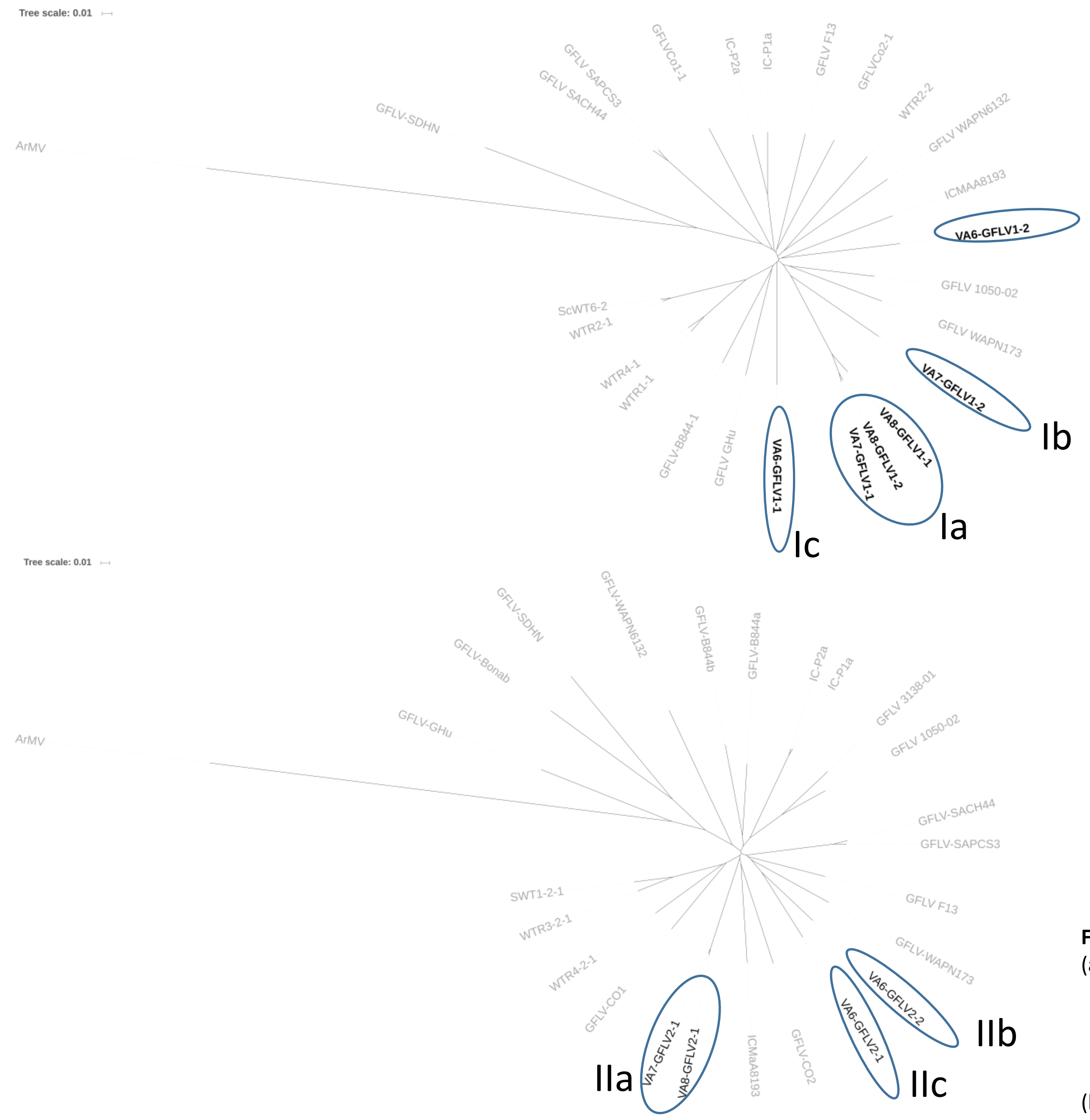

(b)

Number of mismatchs

		1	2	3	4	5	6
VA7-GFLV1-1	1		75	260	856	882	838
VA8-GFLV1-2	2	98,98		208	860	890	838
VA8-GFLV1-1	3	96,46	97,17		661	917	871
VA7-GFLV1-2	4	88,34	88,29	91,00		904	861
VA6-GFLV1-1	5	87,99	87,88	87,52	87,69		842
VA6-GFLV1-2	6	88,59	88,59	88,14	88,28	88,53	

% of nucleotide identity

Number of mismatchs

		1	2	3	4
VA6-GFLV2-1	1		332	422	434
VA6-GFLV2-2	2	91,22		453	461
VA7-GFLV2-1	3	88,84	88,01		37
VA8-GFLV2-1	4	88,55	87,82	99,02	

% of nucleotide identity

Figure S1. Genetic diversity of GFLV sequences from grapevines VA6, VA7 and V8.

(a) Phylogenetic trees based on alignements of sequences corresponding to ORF1 (top panel) and ORF2 (lower panel), were reconstructed with the Neighbor Joining (NJ) algorithm with bootstrapping analyses of 1,000 replicates. Name of sequences from VA6, VA7 and VA8 are written in black (with the first number after GFLV corresponding to ARN1 or ARN2 and the last number corresponding to the molecular variant). The names written in grey correspond to sequences from GenBank. The name of clades is the same as in Figure 4.

(b) Tables displaying mismatch numbers and percentage of nucleotide identity between ORF1 (top panel) and ORF2 (lower panel) nucleotide sequences. Analyses were performed by CLC Workbench software.
