# Supplementary material for: Detection of Multiple Variants of Grapevine Fanleaf Virus in Single Xiphinema index Nematodes
Source: Viruses. 2019 Dec 10;11(12):1139. doi: 10.3390/v11121139 (PMC6950412; doi:10.3390/v11121139)
Supplement: Supplementary file 1 [file viruses-11-01139-s001.zip › FigureS2.pdf]

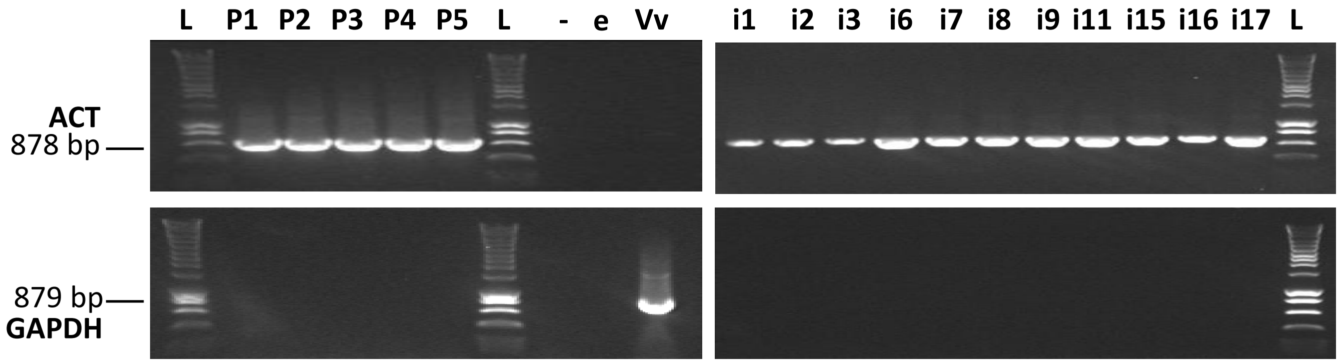

Figure S2. Assessment by RT-PCR of mRNA quality from *X. index* nematodes. Amplicons are targeting actin (ACT) nematode endogene expression (both top panel' gels) and grapevine GAPDH (both lower panel' gels). The size in base pairs (bp) are indicated on the left for each gene tested. P1 to P5 correspond to the five pool of 30 nematodes and i1 to i17 outlined the 11 positive single nematode's extracts. "L" corresponds to ladder; - indicates negative controls; "e" for empty wells and Vv for *Vitis vinifera* GADPH positive control.
