# Supplementary material for: Detection of Multiple Variants of Grapevine Fanleaf Virus in Single Xiphinema index Nematodes
Source: Viruses. 2019 Dec 10;11(12):1139. doi: 10.3390/v11121139 (PMC6950412; doi:10.3390/v11121139)
Supplement: Supplementary file 1 [file viruses-11-01139-s001.zip › FigureS3.pdf]

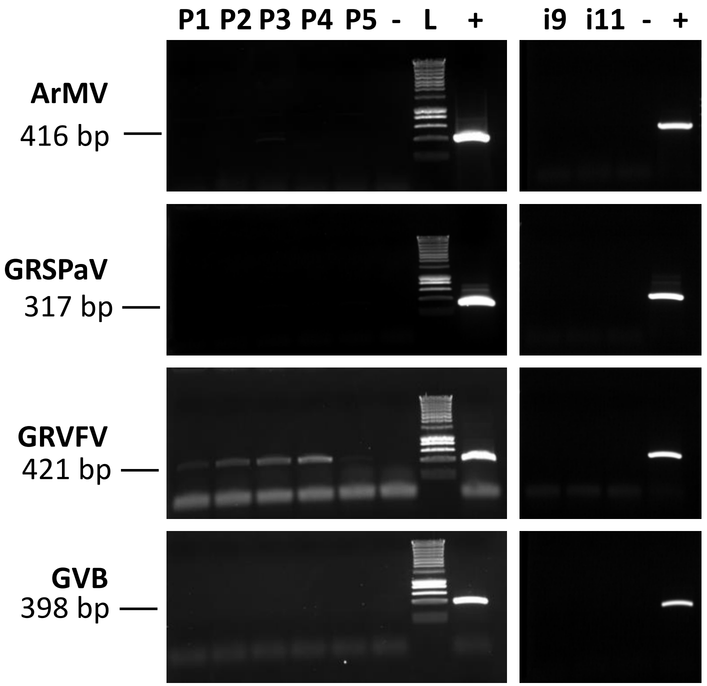

Figure S3. Detection by RT-PCR of viruses other than GFLV in nematodes. Pool of 30 nematodes (P1 to P5) and single nematode (i9 and i11). The size in base pairs (bp) are indicated on the left for each virus tested. L corresponds to ladder, “+” is for positive controls and “-” indicates negative controls.
