# Supplementary material for: Detection of Multiple Variants of Grapevine Fanleaf Virus in Single Xiphinema index Nematodes
Source: Viruses. 2019 Dec 10;11(12):1139. doi: 10.3390/v11121139 (PMC6950412; doi:10.3390/v11121139)
Supplement: Supplementary file 1 [file viruses-11-01139-s001.zip › TableS1.pdf]

Table S1. List of primers.

Organism	Primer name	Product size (bp)	Sequence	Application
Nematode	Nem-act-fwd	878	ATCCAAGCTGTGTTGTCCTT	RT-PCR
	Nem-act-rev		CCGCTCTCGTCGTATTCTTG	RT-PCR
	Actin-nem-qfwd	111	ACACTGTGCCCATCTATGAAG	RT-qPCR
	Actin-nem-qrev		TAACCACGTTCAGTGAGGATTT	RT-qPCR
Grapevine	GAPDH-fwd	879	CGATCCATTCATCACCACTG	RT-PCR
	GAPDH-rev		ATCACACGGGAGCTGTAACC	RT-PCR
	GAPDH-qfwd	122	CAATGAAGGACTGGAGAGGT	RT-qPCR
	GAPDH-qrev		GCCATTCCAGTCAATTTCCC	RT-qPCR
ArMV	RNA2-ArMV-3140-fwd	416	CTTTGGGTGGACTTCTCAGCAT	RT-PCR
	RNA2-ArMV-3540-rev		CTTGGTTTGCACAAGATTCGGAGC	RT-PCR
	RNA2-ArMV-3137-qfwd	105	GGGTGGACTTCTCAGCATTTA	RT-qPCR
	RNA2-ArMV-3242-qrev		GGATTATCTCCCAGGTGAACAG	RT-qPCR
GRSPaV	GRSPaV-8300-fwd	317	ATGARAATGAAAARTTTGCAGCCTTTGA	RT-PCR
	GRSPaV-8620-rev		TTCCAGCGAACAGGCTTAACC	RT-PCR
GRVFV	GRVFV-6180-fwd	421	CCCTTCCARTGGGTTGCTCTCATAA	RT-PCR
	GRVFV-6610-rev		CTGACCATGCCACGRATCATGA	RT-PCR
	GRVFVall-qfwd	141	GAGCTACAAGGTGAATTGCTTCA	RT-qPCR
	GRVFVall-qrev		CGATGGTCATCGCGAGAAGG	RT-qPCR
GVB	GVB-7020-fwd	398	CTCAAGGCTCTTTCGAACTGAGG	RT-PCR
	GVB-7400-rev		CGCATTCCAAACGGAGCTTTACT	RT-PCR
	GVB-qfwd	115	AAACGGTGGTCGGTACATAT	RT-qPCR
	GVB-qrev		GCATTCCAAACGGAGCTTTAC	RT-qPCR
GFLV	RNA1-GFLV-6405-fwd	733	CTRCCTTGTGGRGAYGGGATWGG	RT-PCR/RFLP
	RNA1-GFLV-7140-rev		GGTTATTTRADCTTGGTTATCCCAGTACC	RT-PCR/RFLP
	RNA1-GFLV-q1G5680fwd	122	Vigne et al., 2018*	RT-qPCR
	RNA1-GFLV-q1G5802reva		Vigne et al., 2018	RT-qPCR
	RNA1-GFLV-q1G5802revb		Vigne et al., 2018	RT-qPCR
	RNA2-GFLV-ic2G3131fwda	567	Vigne et al., 2018	RT-PCR/RFLP
	RNA2-GFLV-ic2G3131fwdb		Vigne et al., 2018	RT-PCR/RFLP
	RNA2-GFLV-ic2G3698rev		Vigne et al., 2018	RT-PCR/RFLP

*Vigne, E.; Garcia, S.; Komar, V.; Lemaire, O.; Hily, J.-M.
Comparison of Serological and Molecular Methods With High-Throughput Sequencing for the Detection and Quantification of Grapevine Fanleaf Virus in Vineyard Samples.
Frontiers in Microbiology 2018, 9, doi:10.3389/fmicb.2018.02726.
