# Supplementary material for: Detection of Multiple Variants of Grapevine Fanleaf Virus in Single Xiphinema index Nematodes
Source: Viruses. 2019 Dec 10;11(12):1139. doi: 10.3390/v11121139 (PMC6950412; doi:10.3390/v11121139)
Supplement: Supplementary file 1 [file viruses-11-01139-s001.zip › TableS2.pdf]

Table S2. Relative quantification by RT-qPCR of viruses other than GFLV in grapevines or nematodes.

The vines, virus source plants, are VA6, VA7 and VA8
ΔCt : Ct Gene of Interest - Ct GAPDH grapevine
ΔCt : Ct Gene of Interest - Ct Actin Nematode
rel. Quant: relative quantification compared to GFLV RNA1 accumulation within the same sample
av.: average of positive samples, with ± corresponding to standard error (SE)
N/A: Not applicable
*: not applicable since sample was negative for GFLV

		GFLV	ArMV	GVB	GRVFV
Grapevine	Vines, virus source plants	3/3	1/3	2/3	1/3
	av. ΔCt	-0.0 ± 0.6	3.8	2.4 ± 0.5	2.2
	av. rel. Quant.	1	0.135	0.133 ± 0.009	0.399
Nematode	Pool	5/5	2/5	0/5	5/5
	av. ΔCt	4.8 ± 0.6	13.7 ± 3.4	N/A	10.4 ± 1.3
	av. rel. Quant.	1	0.005 ± 0.005	N/A	0.190 ± 0.167
	Single	9/11	2/11	0/11	1/11
	av. ΔCt	6.7 ± 0.6	11.7 ± 1.0	N/A	0.9
	av. rel. Quant.	1	0.113 ± 0.111	N/A	N/A*
